# Aberrant calcium signalling downstream of mutations in TP53 and the PI3K/AKT pathway genes promotes disease progression and therapy resistance in triple negative breast cancer

**DOI:** 10.20517/cdr.2022.41

**Published:** 2022-06-21

**Authors:** Alex J. Eustace, Min Jie Lee, Grace Colley, Jack Roban, Tim Downing, Paul J. Buchanan

**Affiliations:** ^1^DCU Cancer Research, Dublin City University, Dublin D9, Ireland.; ^2^National Institute Cellular Biotechnology, Dublin City University, Dublin D9, Ireland.; ^3^School of Biotechnology, Dublin City University, Dublin D9, Ireland.; ^4^School of Nursing, Psychotherapy, and Community Health, Dublin City University, Dublin D9, Ireland.

**Keywords:** Triple-negative breast cancer, TP53, PI3K/AKT pathway, calcium

## Abstract

Triple-negative breast cancer (TNBC) is characterized as an aggressive form of breast cancer (BC) associated with poor patient outcomes. For the majority of patients, there is a lack of approved targeted therapies. Therefore, chemotherapy remains a key treatment option for these patients, but significant issues around acquired resistance limit its efficacy. Thus, TNBC has an unmet need for new targeted personalized medicine approaches. Calcium (Ca^2+^) is a ubiquitous second messenger that is known to control a range of key cellular processes by mediating signalling transduction and gene transcription. Changes in Ca^2+^ through altered calcium channel expression or activity are known to promote tumorigenesis and treatment resistance in a range of cancers including BC. Emerging evidence shows that this is mediated by Ca^2+^ modulation, supporting the function of tumour suppressor genes (TSGs) and oncogenes. This review provides insight into the underlying alterations in calcium signalling and how it plays a key role in promoting disease progression and therapy resistance in TNBC which harbours mutations in tumour protein p53 (TP53) and the PI3K/AKT pathway.

## INTRODUCTION

### Triple-negative breast cancer is associated with a worse disease outcome

Breast cancer (BC) is the most common female-specific cancer in the world^[1]^. Triple-negative BC (TNBC) accounts for ~15% of all BC cases and is characterized by a clinically more aggressive disease, which is linked with higher recurrence rates, increased metastatic potential and poorer overall survival^[2-4]^. Unlike oestrogen receptor-positive (ER+) BC and human epidermal growth factor receptor 2 (HER2)-positive BC, TNBC lacks the expression of oestrogen, progesterone and HER2 receptors^[5,6]^. In both ER+ and HER2-positive BC, the expression at the protein and/or gene level of these receptors has been successfully targeted as a cancer treatment with either small molecule inhibitors or monoclonal antibodies^[7]^. The results of these interventions in ER+ and HER2-positive BC have resulted in both improved response rates and overall survival of those BC patients^[8,9]^. In TNBC, the development of targeted approaches to treat the disease lags behind other BC subtypes meaning that TNBC has one of the poorest survival rates of all breast cancer subtypes^[10,11]^.

In TNBC, chemotherapy remains one of the main treatment options for patients, but acquired resistance to chemotherapy remains a significant clinical problem^[12]^. In recent years, advances in treating TNBC patients with novel targeted agents have shown benefits. For example, for patients who have a germline breast cancer gene (BRCA) 1/2 mutation, poly(ADP-Ribose) polymerase (PARP) inhibitors are used to treat TNBC patients in both the adjuvant and metastatic settings^[13-15]^. However, patients with *BRCA* mutations account for only a small percentage of TNBC cases. Immunotherapy offers new hope for TNBC patients: results from the recent IMPASSION study demonstrate that immunotherapy combined with chemotherapy offers benefit to a subset of patients who have elevated programmed death ligand (*PDL*) 1 expression^[16]^. Lastly, tumour-associated calcium signal transducer 2 (TROP2), encoded by the (*TACSTD2*) gene, is a transmembrane glycoprotein expressed in approximately 80% of TNBC. The antibody-drug conjugate (ADC) sacituzumab govitecan, a TROP2-directed antibody and topoisomerase inhibitor drug conjugate, has been approved for the treatment of TNBC by the FDA^[17]^. However, there remains a significant proportion of TNBC patients for whom these therapies offer little benefit.

Calcium (Ca^2+^) is an essential component required for normal cellular function and is involved in the regulation of processes such as metabolism, muscle contraction and phagocytosis as well as cell growth, proliferation and apoptosis^[18,19]^. Local Ca^2+^ concentrations oscillate with varying frequency and amplitude, enabling the induction or modulation of signal transduction and gene transcription^[20-22]^. In line with this, it has become evident that Ca^2+^ supports the functions of key tumour suppressor genes (TSGs) and oncogenes commonly altered in BC, such as tumour protein p53 (TP53), phosphatase and tensin homolog (PTEN) and Phosphatidylinositol-4,5-bisphosphate 3-kinase catalytic subunit alpha (PIK3CA), as well as a number of others reviewed elsewhere^[23]^. Generally, oncogenes promote cellular survival by dampening Ca^2+^ signalling, whereas TSGs induce apoptosis through Ca^2+^ influx^[23]^. Owing to this key role, it is unsurprising that Ca^2+^ is altered downstream of key driver genes, enabling cancer progression and treatment resistance^[18,24,25]^. Consequently, Ca^2+^ represents an area of interest for the development of new drug therapies targeted to specific genomic alternations^[26-28]^. This review outlines in detail the mechanisms of Ca^2+^ modulation and how these mechanisms are altered in specific TNBC cohorts who harbour mutations in TSGs such as TP53 and PTEN and oncogenes such as PIK3CA.

### The role of calcium in cancer

Ca^2+^ plays a key role in cell growth, proliferation and apoptosis through rapid local fluctuations in intracellular calcium (Ca_i_^2+^), as well as long-term genomic changes by regulating signal transduction and gene transcription. In non-excitable cells such as epithelial cells in BC, Ca_i_^2+^ is predominately modulated through store-operated calcium (SOC). Here, store-operated calcium channels (SOCCs) allow the influx of Ca^2+^ into the cytosol due to differential Ca^2+^ concentrations which are established and tightly regulated through the continual action of various pumps and calcium channels^[18,24,29]^. Extracellular Ca^2+^ is maintained at a concentration of 2 mM and cytosolic Ca^2+^ at a concentration of 100 nM^[30]^. In addition, the ER and mitochondria act as Ca^2+^ stores, holding Ca^2+^ concentrations of ~1 mM and ~200 nM, respectively^[31]^. These differential calcium gradients allow for localised changes in Ca^2+^ through the action of calcium channels, which in turn promotes various functions such as signal transduction and gene transcription as well as cellular processes such as proliferation and apoptosis.

SOC is initiated by Ca^2+^ release from the endoplasmic reticulum (ER) through associated calcium channels such as inositol trisphosphate (IP3) receptors (IP3Rs) or ryanodine receptors (RyRs). IP3R activation is initiated by upstream activation of plasma membrane G protein-coupled receptors (GPCRs) and receptor tyrosine kinase (RTK)^[32]^. Following activation of these receptors by their associated ligands, phospholipase C (PLC) enzyme is produced, hydrolysing phosphatidylinositol 4,5-bisphosphate (PIP2) and resulting in the production of diacylglycerol (DAG) and IP3, the latter of which activates IP3R inducing ER Ca^2+^ release^[33]^. In addition, the channel can also be activated by common cellular stresses found in the tumour microenvironment (TME), such as reactive oxygen species (ROS), ER stress, altered cellular energetics, hypoxia and drug treatment^[23]^. Alternatively, RyR releases Ca^2+^ from the ER upon sensing changes in intracellular Ca^2+[34]^. Subsequently, the decrease in store Ca^2+^ is detected by a family of stromal interaction molecule (STIM) channels^[35]^, which mediate store-operated calcium entry (SOCE) primarily through calcium release-activated calcium modulator (ORAI) and occasionally transient receptor potential (TRP) channels at the plasma membrane^[36-38]^. Normal Ca^2+^ concentrations are subsequently restored through Ca^2+^ efflux via the plasma membrane calcium pump (PMCA) and Na^+^/Ca^2+^ exchanger (NCX) along with reabsorption into the ER through the sarco/endoplasmic reticulum Ca^2+^ ATPase (SERCA). In addition, it has been increasingly found that the membrane potential of cancer cells has become more depolarised, and as such voltage-gated calcium channels (VGCC) have also been shown to contribute to calcium entry^[39,40]^. Furthermore, these channels have also been shown to contribute to SOCE in cancer cells^[41]^.

Research has continued to demonstrate that aberrant Ca_i_^2+^ is a common feature in cancer that is able to promote neoplastic transformation and drug resistance^[20,42,43]^. This is mediated through altered calcium channel expression or activity, enabling cancer hallmarks such as migration/invasion, proliferation, survival and apoptotic resistance^[25,27,28,44]^. Typically, increased Ca_i_^2+^ enhances proliferation, while a reduction and faster recovery are linked to apoptotic resistance and decreased sensitivity to chemotherapeutic agents^[45]^. To date, several studies have shown various calcium channel families are altered in BC and linked to tumorigenesis^[31]^. Importantly, this work has also uncovered specific differences in channel expression between BC subtypes^[46]^. For example, IP3R2 and IP3R3 have been found to be upregulated in TNBC tissue compared to luminal subtypes. SOCE is also altered in TNBC through increased ORAI1 and STIM1 expression, which promotes invasion and migration, while in patient samples, it was linked to poorer prognosis^[47,48]^. In contrast, ORAI3 was found upregulated in luminal and HER2 BC^[49]^. SOCE inhibitors in early preclinical studies have demonstrated an ability to inhibit proliferation and migration in MDA-MB-231 TNBC cell lines and reduce tumour growth in TNBC mouse models^[50]^. In addition, Azimi *et al.* (2017) observed that TRPC1 is significantly increased in TNBC compared to all other BC subtypes, resulting in an enhanced epithelial-mesenchymal transition (EMT) phenotype^[51]^. Going forward, this review focuses on the role of Ca^2+^ in supporting TSGs and oncogenes commonly altered in TNBC; however, altered calcium channel expression in BC subtypes has been extensively reviewed elsewhere^[24,46,52]^.

### The role that calcium plays on the impact of TP53 mutations in TNBC

TNBC is a heterogeneous disease, which includes distinct molecular subtypes^[6,53-55]^. This heterogeneity partly explains the limited impact that targeted therapeutic approaches have made in treating the majority of TNBC patients. Part of this heterogeneity is associated with the mutational background of TNBC, which could impact a patient’s response to therapeutic intervention. However, this could provide opportunities for scientists and clinicians to design novel approaches to treat TNBC.

Somatic mutations in the TSG TP53 (located at 17p13.1) occur in ~80% of TNBC cases^[56,57]^. Normally, TP53 is activated due to various stress signals such as DNA damage, hypoxia, ROS, oncogenic activation and cancer treatment^[23]^. In this way, TP53 helps prevent tumorigenesis by regulating biological processes such as apoptosis, cell cycle, DNA repair and senescence^[58]^. Mutations that result in loss of TP53 activity and impair its normal cellular functions have been linked to chemoresistance as well as reduced overall survival^[59-64]^. Most (90%) functionally relevant TP53 mutations produce missense products that have both loss- and gain-of-function features^[65,66]^ and are more resilient to degradation^[57]^. Most known cancer-associated TP53 coding DNA sequence (CDS) changes are within the DNA-binding domain corresponding to exons 5-8 and amino acids 102-292, with > 28% of these nonsynonymous SNPs occurring at eight key sites^[67]^. Many of these originate from aberrant CpG methylation^[67]^. Notably, recurrent TP53 aberrations during cancer growth result in selective sweeps of new clones with independent mutant TP53 proteins^[68]^.

**Figure 1:**
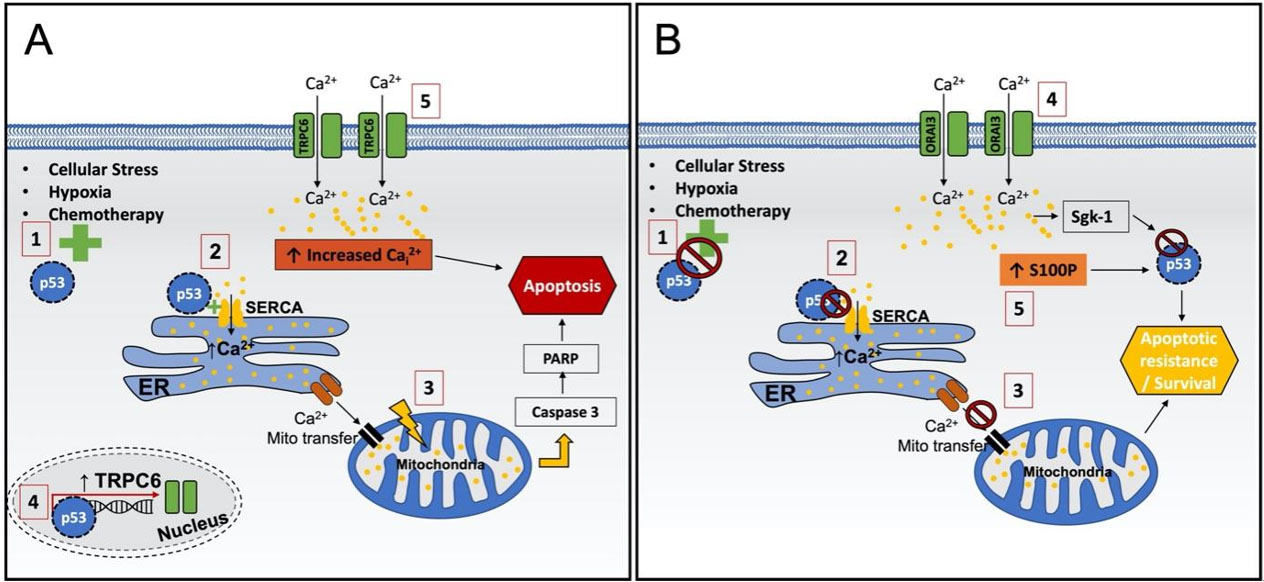
Summary of wild-type and mutant p53 calcium mediated cell death processes. (A) Wild-type p53: (1) induces apoptosis by different cellular stresses such as hypoxia and chemotherapy; (2) mediates an increase in ER Ca^2+ ^through SERCA; (3) promotes ER Ca^2+ ^transfer to the mitochondria resulting in a Ca^2+ ^overloading activated caspase 3 and thus PARP; and (4) mediates apoptosis through an induction in TRPC6 expression and (5) associated Ca^2+ ^influx. (B) (1) Mutations in TP53 result in a loss of function; (2) TP53 fails to induce SERCA; (3) this leads to reduced mitochondrial Ca^2+ ^promoting apoptotic resistance; (4) ORAI3 is increased in TNBC, promoting Ca^2+^-mediated increase in serum and glucocorticoid-induced protein kinase-1 (SGK-1) inducing TP53 degradation and (5) increased S100 calcium-binding protein P (S100P) also induces TP53 degradation. Both enhance cell survival. ER: Endoplasmic reticulum; PARP: poly(ADP-Ribose) polymerase; SERCA: sarco/endoplasmic reticulum Ca^2+^ ATPase.

In relation to Ca^2+^, TP53 is known to have a non-transcriptional role in the cytosol, inducing apoptosis in cells by regulating Ca^2+^ release from the ER^[69,70]^. Recent research has shown that wild-type TP53 localises to ER and mitochondria-associated membranes (MAMs), where it interacts with SERCA increasing Ca^2+^ loading in the ER by enhancing its activity^[71,72] ^[Figure 1A]. In addition, TP53 also promotes ER Ca^2+^ transfer to the mitochondria inducing pro-apoptotic mitochondrial overload, leading to the release of pro-apoptotic factors^[72]^.

Mutant TP53 disrupts these processes, leading to apoptotic resistance and reduced sensitivity to chemotherapy^[69,72,73]^. Here, TP53 fails to induce SERCA activity, reducing mitochondrial Ca^2+^ response, which also reduces caspase 3 and PARP cleavage, thus promoting an anti-apoptotic phenotype^[72] ^[Figure 1B]. In addition, Giorgi *et al.* (2015) demonstrated that wild-type TP53 cells were sensitive to doxorubicin (Adriamycin) treatment, as TP53 mediated an increase in cleavage of PARP and caspase 3, which resulted in reduced cell survival^[72]^. However, in TNBC TP53 mutant MDA-MB 468 cell lines, doxorubicin failed to increase both SERCA activity and Ca^2+^ levels, thus conferring chemotherapy resistance^[72]^. This critical role of TP53 in promoting pro-apoptotic Ca^2+^ in treatment sensitivity was further confirmed by Giorgi *et al.*, who demonstrated that overexpression of SERCA or mitochondrial calcium uniporter (MCU) restored treatment sensitivity^[74]^. This effect was lost when Ca^2+^ levels were reduced by chelation, highlighting how Ca^2+^ is integral to the function of TP53^[74]^. In addition, mouse xenograft tumours established using mouse embryo fibroblast (MEF) H-ras induced cells were observed to double in size with TP53 loss compared to wild-type controls, corresponding with an observed decrease in Ca_i_^2+^ Activity^[74]^.

Other calcium channels such as TRP have also been linked to TP53 pro-apoptotic Ca_i_^2+ ^changes in BC. TRP channels promote calcium entry at the plasma membrane following activation by PLC, DAG or store release from IP3R channels^[75]^. These channels support store refilling via SOCE through the interaction with ORAI and STIM^[76]^. A novel anti-neoplastic organic derivative GaQ3 (which has undergone phase 1 clinical trials in patients with solid tumours) was demonstrated to induce apoptosis by promoting TP53 expression through elevated Ca_i_^2+^ concentrations^[77,78]^. The key role of Ca^2+^ in this mechanism was confirmed using the Ca^2+^ quencher TMB-8, which prevented an increase in Ca_i_^2+^, leading to an inhibition of TP53-mediated apoptosis. Further work found that this increase in Ca_i_^2+^ was mediated by the binding of TP53 to the *TRPC6* promoter, resulting in the overexpression of TRPC6, enhancing Ca^2+^-dependent apoptosis in MCF-7 BC cells^[78]^. A putative TP53 binding site on the *TRPC6 *promoter was also identified by bioinformatic analysis using the Mat Inspector module of the genomatix database, suggesting TP53 is a potential regulator of TRPC6^[78]^. Thus, it was identified that the Ca_i_^2+^ released through TRPC6 is mediated by TP53 and plays a major role in promoting apoptosis in a range of cancer cell line models including MCF-7^[77,78]^.

Research in a range of BC subtypes including TNBC has also observed that altered SOC channel expression is linked to apoptotic and chemotherapy resistance through the TP53 pathways^[23,79]^. A recent study demonstrated through bioinformatic analysis that ORAI3 was elevated in patients with poor response or residual disease following chemotherapy treatment, which was also predictive of poor patient outcomes^[80]^. Increases in ORAI3 expression induced resistance to the chemotherapeutic drugs cisplatin, 5-FU and paclitaxel by reducing apoptosis and increasing survival of T47D and MCF7 BC cell lines^[80]^. These effects were found to be mediated by ORAI3, promoting a decrease in TP53 and cyclin-dependent kinase inhibitor 1A (p21). This was confirmed by the removal of extracellular Ca^2+^ and/or Orai3 functionality, which resulted in an increase in TP53 expression and reduced chemoresistance. Interestingly, Hasna *et al.* (2018) also discovered that the Phosphoinositide 3-Kinase (PI3K)-Protein Kinase B (AKT) pathway was also induced by the observed increase of ORAI3 expression in chemoresistant cells^[80]^. PI3K inhibitors partially inhibited resistance induced by TP53 expression. This chemoresistant effect was mediated by PI3K induction of the SGK-1, leading to TP53 degradation via MDM2 proto-oncogene (Mdm2) and Nedd4-like E3 ubiquitin-protein ligase (Nedd4-2). Prior to this work, Brickley *et al.* (2013) highlighted a link between SOCE and SGK-1 in TNBC cell lines^[81]^. SOCE activation induced the expression of SGK-1 following exposure to cellular stress. This mechanism appeared cytoprotective, as siRNA targeting SGK-1 under the same conditions increased apoptosis.

Proteins such as S100P are also linked to TP53, where their expression was found to be altered in BC, enabling drug resistance^[82]^. Specifically, the expression of S100P is elevated in TNBC and linked to chemotherapy resistance and poor survival^[83-86]^. Gibadulinova *et al.* identified reduced phosphorylation and activity of TP53 in response to DNA damage following S100P binding in a range of cancer cell lines including BC cell lines MCF7 and T47D^[87]^. When bound to TP53 protein, S100P promotes cell survival and resistance towards anticancer drugs such as paclitaxel and cisplatin, evading senescence and thus promoting cancer progression^[87]^.

Until recently, it has been challenging to develop drugs that can target TP53 for cancer treatment^[88]^. This is primarily because TP53 expression is lost in ~10% of cases, and in the other cases, the function of the mutant protein is changed. However, advances in drug design have enabled both scientists and clinicians to develop drugs such as Eprenetapopt (APR-246) and COTI-2, which aim to reactivate TP53. These TP53 reactivators have been tested in preclinical studies in TNBC^[89]^ but also in clinical trials of TP53-mutant myelodysplastic syndromes (MDS)^[90]^ and solid tumours (NCT02433626). Further investigation is warranted to ascertain if these drugs impact any of the calcium signalling mechanisms mentioned previously.

Taken together, there is compelling evidence that Ca^2+^-dependent TP53 apoptosis and associated mechanisms contribute to poor outcomes in TNBC and resistance to chemotherapy. Consequently, it highlights calcium channels as a therapeutic target to modulate Ca^2+^ concentrations in mutant TP53 TNBC, which, through TP53 restoration or calcium channel activation drugs, could lead to improved responses to existing treatments and better overall patient survival.

### Calcium signalling in PI3k/AKT and PTEN pathways in TNBC

PI3K/AKT oncogenic signalling through pathway activation is linked to cell survival, growth and apoptosis^[91]^. Alterations in this pathway are common in TNBC and are associated with poorer outcomes and treatment resistance^[92]^. Up to 30% of TNBC tumours harbour aberrations in the oncogenic PI3K/AKT pathway through mutation of either AKT serine/threonine kinase 1 (AKT1) or PIK3CA^[93,94]^. The loss of wild-type TP53 regulation of AKT (located at 14q32.33), PIK3CA (at 3q26.32) and PTEN (at 10q23.31) has consequences for the PI3K-AKT pathway. PIK3CA encodes an alpha subunit of Phosphatidylinositol 3-Kinase (PI3K). PTEN is another TSG whose mutation can mimic the effects of mutant TP53^[95]^. PTEN is a negative regulator of the PI3K/AKT pathway through downregulation of phosphorylated AKT, and it is also commonly altered in TNBC^[94,96]^.

TP53, AKT, PIK3CA and PTEN changes together constitute the genetic basis of the majority of TNBC cases and initiate the initial stages of tumour development^[97]^. Mutations at these four genes may be oncogenic on their own, or may occur in tandem with other TSG mutations: PIK3CA, PIK3R1 (encoding regulatory protein p85α) and PTEN commonly co-occur in BC^[98]^. Changes at AKT, PIK3CA and PTEN are much less common than those at TP53^[61]^. PIK3CA mutations are typically missense and constitute ~10%-18% of cases, and in ~4% of cases, PIK3R1 may be mutated^[99,100]^ - often, PIK3CA and PIK3R1 co-occur independently^[101]^. PTEN is mutated in ~6%-7% of cases and AKT1 is rarer at ~3%^[100]^. Although all 4 genes may have cancer-related SNPs, TP53 tends not to have other mutation types, in contrast to AKT and PIK3CA that can be amplified or possess CNVs; additionally, PTEN tends to be inactivated, often by deletion.

**Figure 2:**
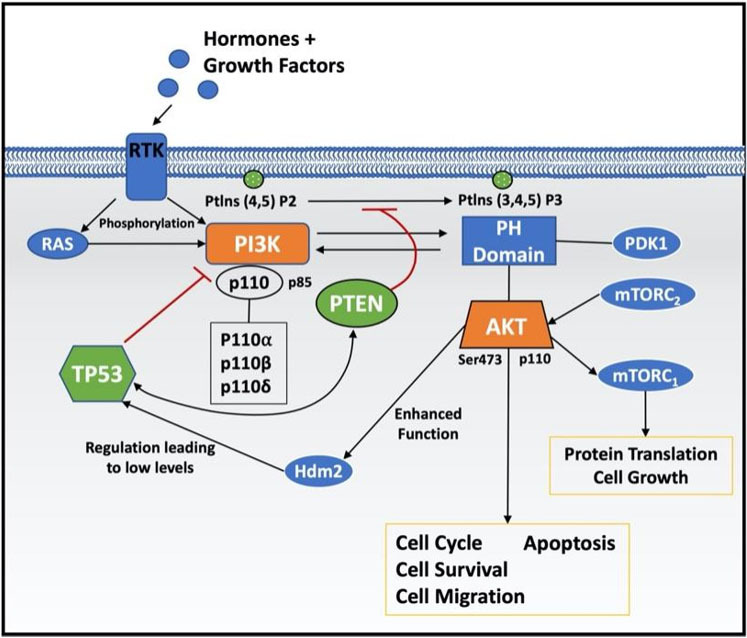
The role of TP53 in the PI3K/AKT signalling pathway. RTKs are activated by hormones and growth factors, leading to the recruitment and phosphorylation of PI3K. PDK1 is then recruited to AKT at the PH domain. Cell cycle, survival, migration and apoptosis regulation occur as a result of AKT signalling pathway activation. AKT is also activated by mTORC_2_ and mTORC_1_ is activated by phosphorylated AKT, promoting protein translation and cell growth. Hdm2 regulates P53 in normal healthy cells, resulting in low levels. P53 regulates PTEN, inhibiting PI3K phosphorylation and AKT activation. AKT may enhance the function of Hdm2 and PTEN may prevent P53 degradation via AKT pathway inhibition. P53 binds to PI3K to inhibit PIK3CA and mutations in TP53 may result in PIK3CA hyperactivation and subsequent PI3K signalling pathway activation. AKT: Protein kinase B; PTEN: phosphatase and tensin homolog.

There is an established link between the PI3K/AKT pathway and TP53 [Figure 2]. RTKs are usually activated and phosphorylated by growth factors and hormones. PI3K, consisting of p110 (α,β,γ,δ) and p85, is recruited to the RTK leading to phosphorylation of PtdIns (4,5) P2 to PtdIns (3,4,5) and subsequent recruitment of PDK1 to AKT at the PH domain. PDK1 is reliant on activation by PI3K. Activation of the AKT signalling pathway results in regulation of the cell cycle, promoting cell growth, survival and migration and inhibiting apoptosis^[102]^. mTORC_2_ activates AKT, thus leading to cell proliferation and survival. mTORC_1_ is activated by phosphorylated AKT and promotes protein translation and cell growth^[103,104]^. TP53 has three primary domains - transactivation domain, DNA-binding domain and tetramerization domain^[105]^. TP53 is regulated by the MDM2 proto-oncogene (Hdm2) protein via binding and physical separation from target genes, resulting in low levels of TP53 in normal cells. TP53 regulates PI3K via binding and inhibition of PIK3CA, which encodes p110α, and reduces AKT activation. Mutations in TP53 may lead to hyperactivation of PIK3CA and the PI3K signalling pathway^[99]^. Studies have demonstrated the regulatory effects of TP53 on PTEN via the functional TP53 binding site present within the PTEN promoter region, resulting in inhibition of PtdIns(4,5)phosphorylation and subsequent AKT activation. AKT can enhance the function of Hdm2 via phosphorylation, and evidence suggests that PTEN may prevent the degradation of TP53 through suppression of AKT activation^[106]^. PTEN has also been identified as a stabiliser of TP53 by way of physical association between the two, conferring protection from degradation to TP53. Despite these stabilising functions, deletion of PTEN and subsequent activation of the PI3K pathway have been shown to activate TP53, with significantly increased TP53 expression present in PTEN-/- cells^[106]^.

PTEN has also been identified as a stabiliser of TP53 by way of physical association between the two, conferring protection from degradation to TP53. Despite these stabilising functions, deletion of PTEN and subsequent activation of the PI3K pathway has been shown to activate TP53, with significantly increased TP53 expression present in PTEN-/- cells^[106]^.

TP53 can regulate cell survival via inhibition of the PIK3CA as well as the PI3K/AKT pathway, independent of PTEN. This transcriptional downregulation of PIK3CA via TP53 has been demonstrated in head and neck cancer cell lines, where the PTEN protein was not detected and induction of TP53 resulted in decreased PIK3CA expression and reduced AKT phosphorylation, indicating the transcriptional regulatory effects of TP53 on PIK3CA^[107]^. A study investigating the mechanisms involved in the regulation of PIK3CA transcription via TP53 in ovarian cancer described two promoters present on the *PIK3CA* gene that have been shown to directly bind TP53 resulting in transcriptional inhibition. It was found that suppression of TP53 resulted in increased levels of p110α transcripts, proteins and PI3K signalling pathway activity, while overexpression of TP53 resulted in decreased levels of p110α protein^[108]^. Loss-of-function mutations in TP53 correlate with hyperactivation of PIK3CA and the PI3K pathway, resulting in worse prognoses for head and neck squamous cell carcinoma patients^[99]^.

**Figure 3:**
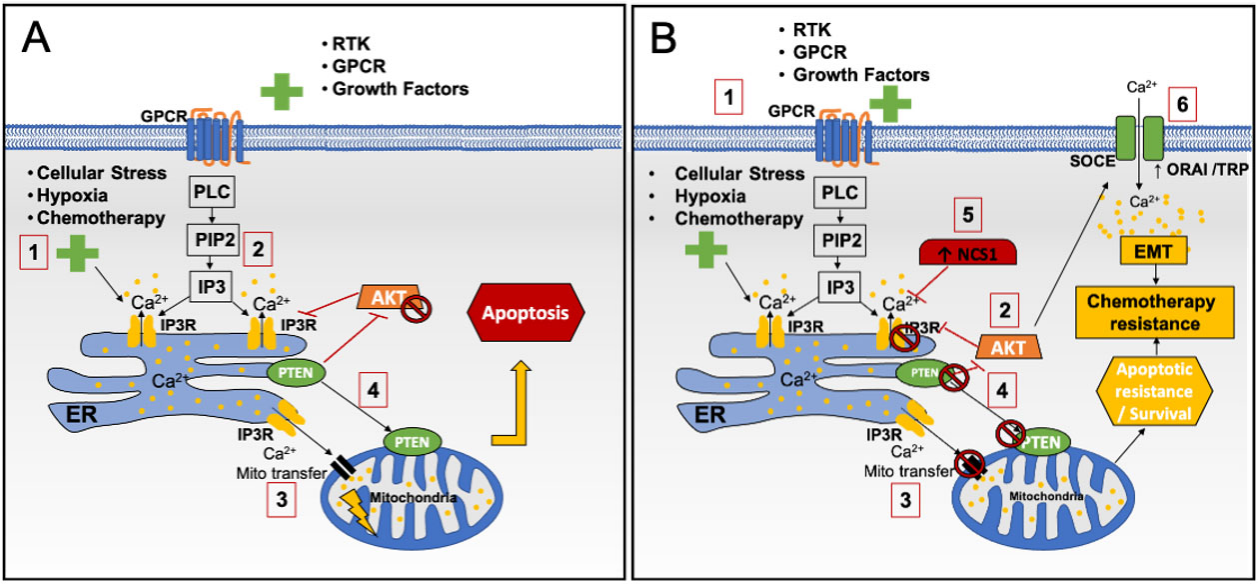
Role of calcium store release via IP3R in mediating apoptosis and its regulation via PI3K/AKT pathways. (A) (1) G protein-coupled receptors (GPCR), Receptor tyrosine kinase (RTK) and others, along with cellular stress, can induce PLC, PIP2 and IP3, leading to activation of IP3R channels on the ER, promoting Ca^2+^ store release. (3) This mediates Ca^2+^ transfer to the mitochondria inducing apoptosis. (4) PTEN also plays a role in mediating this pathway in part by blocking anti-apoptotic oncogene AKT. (B) (1) Activation of oncogenes such as AKT inhibits IP3R Ca^2+^ release from ER stores under chemotherapy and GPCR activation. (2) This is achieved by phosphorylating IP3R and (3) reducing Ca^2+^ transfer to the mitochondria, enabling apoptotic resistance. (4) PTEN loss is common in TNBC, enabling AKT signalling. (5) NCS-1 is increased in TNBC, promoting IP3R inactivation as well. (6) SOCE is mediated after store release via IP3R; channels such as ORAI and TRP that are activated have increased expression in TNBC and are linked to promoting EMT and thus chemotherapy resistance. RTK: Receptor tyrosine kinase; Orai: calcium release-activated calcium channel protein; PLC: phospholipase C; GPCR: G protein-coupled receptors.

Research has demonstrated that the PI3K/AKT pathway can modulate Ca^2+^ release from ER stores via interaction with IP3R^[23] ^[Figure 3A]. This calcium channel is found on the ER membrane, where it controls the frequency and amplitude of Ca^2+^ oscillations to either promote cell survival or induce apoptosis^[109]^. Under hypoxia conditions or chemotherapy treatment, IP3R promotes Ca^2+^ transfer to the mitochondria, inducing the release of pro-apoptotic factors^[23]^. However, the anti-apoptotic protein AKT has been shown to phosphorylate IP3R, preventing ER Ca^2+^ transfer to the mitochondria and inhibiting apoptosis^[110,111] ^[Figure 3B]. This, in turn, enables resistance to chemotherapy and promotes survival during hypoxia^[110,111]^. The PI3K/AKT pathway is also negatively regulated by the TSG PTEN^[112]^, which is commonly lost or mutated in TNBC and associated with poorer prognosis as well as treatment resistance^[113,114]^. PTEN is known to localise at the ER and reduce AKT-induced phosphorylation of IP3R, thus promoting ER Ca^2+^ release. In addition, it also associates with MAM and regulates the transfer of Ca^2+^ to the mitochondria, enabling Ca^2+^-mediated apoptosis^[115]^. In PTEN mutant BC, IP3R becomes phosphorylated and inactivated by AKT, decreasing Ca^2+^ release from the ER and associated transients to the mitochondria, reducing the sensitivity to Ca^2+^-mediated apoptosis^[115]^. This work highlights a potential link between PTEN loss and AKT activation, supporting treatment resistance and disease progression via deactivated IP3R mediated apoptotic resistance.

In relation to IP3R, its ability to mediate Ca^2+^ release from ER can be finetuned through interaction with calcium binding proteins such as neuronal calcium sensor-1 (NCS-1)^[116]^. Recent studies in TNBC cells have shown that overexpression of NCS-1 increases Ca^2+^, promoting survival^[117]^. Furthermore, this mechanism was linked to paclitaxel resistance, where NCS-1 binding to IP3R promotes drug resistance^[116,118] ^[Figure 3B]. Interestingly, researchers found IP3R2 and IP3R3 expression was elevated in breast tumours compared to adjacent normal cells^[119]^. Furthermore, Singh *et al.* (2017), analysing MCF-7 cells, demonstrated that IP3R inhibition is able to induce autophagy and subsequent cell death, an effect not observed in non-tumorigenic cells^[119]^. In addition, IP3Rs are also associated with the nuclear envelope and nucleoplasmic reticulum, where I3PRs can also regulate Ca^2+^ levels within the nucleus to promote cell proliferation^[120,121]^. In TNBC, reducing nuclear IP3R Ca^2+^ by chelation decreased cell proliferation and induced tumour necrosis^[122]^. Overall, this research identified that IP3R can impact both PI3K/AKT dependant and independent pathways.

Ca^2+^ store release via IP3R, which is modulated by PI3K-AKT/PTEN pathways, has an indirect contribution to drug-resistant mechanisms by association with Ca^2+^ binding proteins. For example, Ca^2+^/calmodulin-dependent kinase (CAMKK) is essential for AMP-activated protein kinase (AMPK) induction following activation by epidermal growth factor (EGF) in TNBC cell lines^[123]^. AMPK is an essential regulator of AKT activation under cellular stress such as drug treatment and hypoxia^[123]^. Ca^2+^ is a requirement for this mechanism as its chelation using BAPTA-2AM completely inhibits AMPK-induced phosphorylated AKT. Specifically, AMPK phosphorylates s-phase kinase-associated protein 2 (Skp2) at S256, triggering the formation of an E3 ligase complex that promotes activation of AKT^[123]^. This outlined mechanism was shown to promote cell survival and apoptotic resistance. In BC patient samples, Skp2 activation correlates with increased AKT and AMPK expression and is associated with poor survival outcomes. In addition, Han *et al.* (2018) found that targeting AMPK-Skp2 in TNBC MDA-MB-231 cell lines increased their sensitivity to anti-EGF receptor-targeted therapy^[123]^. Furthermore, this study also showed in the same TNBC cell lines that this mechanism is not only induced by EGF but also hypoxia, promoting survival under such conditions. Hypoxia is a common characteristic in the tumour microenvironment (TME), which is also linked to chemotherapeutic resistance^[73]^.

SOCE mediated through ORAI and STIM as well as TRP enables the refiling of ER stores following PI3K-IP3R induced release, which supports signal transduction. Both ORAI and STIM have been shown to be upregulated in TNBC, enabling disease progression and promoting treatment resistance^[124]^. Using TNBC MDA-MB-231 cell lines, Bhattacharya *et al.* further demonstrated that (in the presence of phosphorylated AKT) ORAI3- and STIM1-mediated Ca^2+^ promotes the induction of Snail family transcriptional repressor (SNAIL)^[125]^. Induction of SNAIL expression is known to mediate EMT, a cellular phenotype linked to drug resistance^[126,127]^. In addition, TRPC1 has also been shown to play a role in EMT of TNBC via regulation of hypoxia-inducible factor 1 alpha (HIF-1α) and AKT signalling in PTEN-deficient BC cells^[51]^. Azimi *et al. * also noted that TRPC1 has a prognostic value in basal BC a molecular subtype associated with TNBC^[51]^. Specifically, high TRPC1 expression was associated with significantly poorer patient relapse-free survival^[51]^. Furthermore, it was noted that TRPC1 can induce EMT and promote chemotherapy resistance in MDA-MB-468 TNBC cell lines through the upregulated ATP-binding cassette, subfamily C, member 3 (ABCC3), a multidrug resistance ATP-binding cassette (ABC) transporter^[128]^. In addition, this channel, alongside ORAI1 and IP3R2, has been found to be upregulated following doxorubicin treatment in MDA-MB-231 TNBC cells^[129]^.

TRPC5 is another family member whose overexpression is linked to chemotherapy resistance by induction of a protective autophagy mechanism which promotes cancer cell survival^[130]^. Adriamycin (ADM/doxorubicin) was found to induce an increase in Ca_i_^2+^ which was reduced with siRNA targeting of TRPC5. Exploration of the underlying pathway discovered that Ca^2+^ mediated through TRPC5 could activate CaMKKβ, AMPKα and mammalian target of rapamycin (mTOR), which resulted in the induction of autophagy^[130]^. As noted above, AMPK modulates AKT activation, which can regulate ER store release, and this can then facilitate chemotherapy resistance. In the study by Zhang *et al.* (2017), targeting the TRPC5-mediated CaMKKβ/AMPKα/mTOR pathway increased sensitivity to ADM^[130]^. Furthermore, analysis of patient samples via a tissue microarray (TMA) found that TRPC5 was significantly increased in patient samples post chemotherapy treatment when compared to pre-treated samples. Supporting the role of TRPC5 in chemotherapy resistance, studies have linked TRPC5 expression to the induction of expression of the multidrug resistance protein 1 (MDR1) in TNBC cells^[18]^.

Because of the large number of TNBCs that have an activated PI3K/AKT pathway, it is unsurprising that early phase clinical trials of both PI3K inhibitors such as buparlisib^[131,132]^ and AKT inhibitors such as capivasertib^[133] ^and ipatasertib^[134]^ have been conducted in this setting. Interestingly, these trials have reported clinical activity and benefits to patients. The identification of key biomarkers of response to these treatments and their study in combination with chemotherapy or other targeted therapies warrants further investigation.

Furthermore, as discussed above, there is a strong link between Ca^2+^ signalling and those TNBC cancers that have a TP53 mutation and/or activation of the PI3K/AKT pathway. Activation of the PI3K/AKT pathway can be achieved through either mutation of PIK3CA/AKT1 or loss of expression of PTEN. Our review highlights that several calcium channels associated with Ca^2+^ release from ER and subsequent SOCE are linked to the activation of the PI3K/AKT pathway. Aberrations in the PI3K/AKT pathway disturb Ca^2+^ handling and enable escape mechanisms such as cell survival and apoptotic resistance, thus promoting chemotherapy resistance.

### Current approaches to target calcium channel signalling in cancer

The role of calcium channels in mediating therapy resistance through interplay with genomic alternations in TNBC highlights calcium channels as potential targets for personalized medicine approaches. The study of calcium channel blockers (CCBs) is an area of intense research^[26-28,135]^. Several calcium blocking drugs have been developed which can modulate calcium channels and be exploited as cancer treatments^[27,43,135,136]^. Calcium channels provide a plethora of targets to modulate Ca_i_^2+^, and ion channel targeting drugs are currently the second largest group of FDA-approved drugs, therefore providing a bank of new drugs which can be repurposed for the treatment of cancer^[137]^.

Several studies have identified existing FDA-approved drugs that can modulate store-operated release and entry through key channels such as ORAI, STIM, TRP and IP3R, as outlined herein, thus representing potential future therapeutics for the tackling disease progression and chemotherapy resistance in the TNBC cohorts outlined. For example, an extensive screen of 1118 unique FDA-approved drugs discovered 11, mainly cardiac glycosides, which have the ability to modify Ca^2+^ signalling, promote Ca^2+^-calmodulin kinase (CamK) activity and reverse the suppression of TSGs such as secreted frizzled related protein 1 (SFRP1), tissue inhibitor of metallopeptidase-3 (TIMP-3) and WNT inhibitory factor 1 (WIF-1), leading to cell death of colorectal cells^[26]^. Other similar studies identified the ability of the immunosuppressant drugs to regulate SOCE, namely, leflunomide used to treat arthritis and teriflunomide used for multiple sclerosis^[138]^. In addition, the drug eflornithine, also known as DFMO, has been found to reduce SOCE and promote a decrease in cell proliferation and cell death resistance in colorectal cancer^[139]^. Eflornithine has been found to inhibit TRPC1 expression, which, as outlined above, plays a role in AKT signalling in PTEN deficient TNBC.

Nonsteroidal anti-inflammatory drugs (NSAIDs) are commonly prescribed anti-inflammatory, anti-pyretic drugs that have demonstrated anti-cancer activity^[140]^. The metabolite of aspirin, salicylate, has been found to inhibit SOCE and mediate mitochondrial uncoupling, reducing mitochondrial Ca^2+^ uptake and leading to a decrease in the proliferation of colon cancer cells^[141]^. Furthermore, other NSAIDs such as ibuprofen and indomethacin have also been shown to modulate SOCE through STIM1, which decreased colorectal cancer growth^[142]^. The CCB mibefradil, previously used to treat hypertension, can also inhibit SOCE by blocking ORAI channels, promoting cell apoptosis and cell cycle arrest^[143]^. In addition, a potent synthetic oestrogen used in the treatment of prostate and breast cancer, diethylstilbesterol, can inhibit SOCE via TRPM7 channels^[144,145]^. The potential to target SOCE modulation for disease benefit has led to the development of a number of drugs, such as CM2489, which has entered into phase 1 clinical trial for psoriasis; if successful, it could prove useful for TNBC treatment^[146]^. CM2489 is a first-in-class calcium release-activated calcium (CRAC) channel inhibitor that targets ORAI channels. Lastly, IP3R channels appear to be a key target in PI3K/AKT pathways mediating chemoresistance; both caffeine and heparin have been shown to inhibit these channels and could hold future potential as novel treatments in combination with existing anti-cancer agents^[147,148]^.

## CONCLUSION

### Targeting calcium channel signalling and key oncogenic pathways is a novel therapeutic approach to treating TNBC

TNBC is characterized as an aggressive form of BC associated with poor patient outcomes. For the majority of patients, there is a lack of approved targeted therapies. TNBC has frequent genomic alternations in TP53 and the PI3K/AKT pathway. This review provides insight into the underlying alterations in Ca_i_^2+^ mediated through calcium channels and how this plays an important role in promoting disease progression and therapy resistance in TNBC harbouring mutations in key TSG and oncogenes. Specifically, TNBC with mutant TP53 is associated with a loss of Ca^2+^ store release via SECRA, leading to reduced mitochondrial Ca^2+^ loading; this, in turn, promotes cell survival and apoptotic resistance, thus enabling treatment resistance. In addition, I3PR-mediated ER store release and SOCE modulation are altered by PI3K/AKT activation, promoting apoptotic resistance. Consequently, the outlined evidence highlights calcium channels as therapeutic targets to modulate altered Ca_i_^2+^ downstream of common genomic alterations in TNBC.

This promise is supported by the fact that a growing number of calcium targeting drugs are under development, as well as a number of existing FDA-approved drugs. However, owing to the complexity of calcium signalling, its ubiquitous nature and differential expression between cell types and genomic alterations, care needs to be exercised when targeting Ca^2+^ signalling. It may be that targeting Ca^2+^ signalling, whilst beneficial for one cancer, could be detrimental for another. Overall, this review highlights Ca^2+^ electroporation as an emerging area in TNBC that could hold significant potential as disease biomarkers as well as future therapeutics when combined with treatments that can inhibit the PI3K-AKT pathway or reactivate TP53 expression.

## DECLARATIONS

### Copyright

© The Author(s) 2022.
